# Dynamic changes of soluble ST2 levels predicted fatality and were involved in coagulopathy in dengue fever in the elderly

**DOI:** 10.1371/journal.pntd.0007974

**Published:** 2019-12-26

**Authors:** Chih-Cheng Hsieh, Ming-Yuan Hong, Tzong-Shiann Ho, Ching-Chuan Liu, Guey-Chuen Perng, Chia-Chang Chuang

**Affiliations:** 1 Division of Critical Care Medicine, Department of Internal Medicine, National Cheng Kung University Hospital, College of Medicine, National Cheng Kung University, Tainan, Taiwan; 2 Department of Emergency Medicine, National Cheng Kung University Hospital, College of Medicine, National Cheng Kung University, Tainan, Taiwan; 3 Department of Pediatrics, National Cheng Kung University Hospital, College of Medicine, National Cheng Kung University, Tainan, Taiwan; 4 Institute of Basic Medical Science, College of Medicine, National Cheng Kung University, Tainan, Taiwan; Fundacao Oswaldo Cruz, BRAZIL

## Abstract

**Background:**

Severe dengue virus (DENV) infection involves plasma leakage and vascular collapse, and leads to significant morbidity and death. Serum soluble ST2 (sST2 [interleukin (IL)-1 receptor like-1 protein: IL-1-RL-1]) levels are high in pediatric cases of DENV infection, and the disease progresses. However, the correlation between serum sST2 levels and the outcomes of DENV infection in the elderly (≥65 years) is unclear. We thus explored the mechanisms of serial sST2 level changes involved in the coagulopathy and bloodstream infections of elderly patients in Taiwan’s 2015 DENV outbreak.

**Methods:**

This retrospective study was done in a tertiary medical center in southern Taiwan during the outbreak. All DENV-infected patients who, between July 1, 2015, and December 31, 2015, provided a written informed consent for at least two blood sample analyses were enrolled and reviewed. The serum levels of sST2 were quantified. ΔsST2 is defined as the “changes of sST2 levels in serially paired samples”. Receiver operating characteristic (ROC) curve and area under the ROC curve (AUC) analyses were used to evaluate the prognostic ability of ΔsST2.

**Results:**

Forty-three patients with DENV infection were enrolled. Mean patient age was 75.0 ± 12.2 years and the case fatality rate was 44.2% (19/43). Significantly more non-survivors than survivors had increased ST2 level (78.9% vs. 12.5%, *p* < 0.001). The AUC value for serum ΔsST2 level was 0.857 for predicting DENV fatality. Moreover, patients given frozen fresh plasma (FFP) transfusions were significantly (*p* = 0.025) more likely to have higher serum ST2 level changes than were those who had not. DENV-infected patients with early bloodstream infections (BSIs) seemed to have higher ST2 levels than those who did not have BSIs.

**Conclusions:**

Serum ST2 levels increased in the elderly (≥ 65 years of age) with DENV infection. The changes in serum sST2 levels might be a critical indicator of DENV infection severity for the elderly; sST2 is an important modulator of coagulopathy in severe DENV infections.

## Author summary

Dengue virus (DENV) infection is a mosquito-borne disease that annually affects at least 50 million people worldwide. The cytokine response during DENV infection is mercurial and abstruse. IL-1 receptor-like-1 (IL-1R-L-1 [aka ST2]) protein is associated with the severity of DENV infection, and elevated cytokine levels are important early predictors of dengue hemorrhagic fever and dengue shock syndrome. ST2, a member of the interleukin-1-receptor/toll-like receptor (TLR) superfamily, is an important biomarker of severe forms of pediatric DENV infection. We first confirmed a similar trend in the elderly cohort. Serial changes of soluble ST2 (sST2) levels were a more reliable predictor for dengue fatality than a single measurement was. Patients given fresh frozen plasma (FFP) transfusions had significantly higher serum ST2 levels than those who had not been given FFP transfusions. SerumsST2 might be necessary for modulating coagulopathy in severe DENV infections. The exact molecular mechanism and the optimal timing for sST2 testing need further investigation.

## Introduction

Dengue virus (DENV) infection annually affects at least 50 million people worldwide and causes lethal complications [1]. DENV infection can be subclinical or present with severe clinical manifestations like dengue hemorrhagic fever (DHF) or dengue shock syndrome (DSS) [2]. In Taiwan’s 2015 DENV outbreak, most patients were elderly (≥ 65 years) and more often presented with atypical signs and symptoms than pediatric patients did [3]. We [4] and another study [5] found that prolonged activated partial thromboplastin time (APTT) was an independent predictive factor for in-hospital fatality in this elderly cohort. However, the exact mechanism of coagulopathy remains unclear. We also determined [4, 6] that some DENV-infected patients in this outbreak had fulminant clinical deterioration when they had bloodstream infections (BSIs).

The immune-mediated response to DENV infection includes cytokine and chemokine expression [7], T-lymphocyte activation [8], and a decline in vascular endothelial growth factor receptor 2 (VEGFR2) expressions [9]. DHF is clinically characterized by increased vascular permeability, plasma leakage, thrombocytopenia, and coagulopathy [10]. The increase in circulating viruses activates T cells, which then release disproportionately large amounts of cytokines; this leads to plasma leakage and coagulation derangements [11]. Many cytokines—e.g., tumor necrosis factor (TNF)-α, interferon (IFN)-γ, interleukin (IL)-6, IL-8, IL-10, IL-1 receptor-like-1 (IL-1R-L-1) protein, and macrophage migration inhibitory factor—are correlated with the severity of DENV infection [12–15], and elevated levels of these cytokines are important early predictors of DHF and DSS [16].

Suppression of tumorigenicity-2 (ST2), a member of the IL-1R/Toll-like receptor (TLR) superfamily [17], is an important biomarker of severe forms of pediatric dengue [18]. There are three forms of ST2: ST2L (longer membrane anchored), ST2V (membrane bound variant), and sST2 (shorter release soluble). The only known ligand of ST2 is IL-33. When IL-33 binds to ST2V, it activates transcription factors NF-κB and AP-1 and then induces the release of proinflammatory cytokines [19]. ST2L co-stimulates an optimal T-helper (Th2)-type response and downregulates macrophage-dependent inflammation, which is also induced by sST2 [20]. Although IL-33 uses the IL-1 receptor ST2, and is an endogenous proinflammatory danger signal, known as “alarmin”, which activates neighboring immune cells after infection or a trauma, which, in turn, upregulates inflammatory responses, but the measurable IL-33 levels in severe pediatric dengue do not change [18, 19]. Recent studies [16] of serial samples from young DENV-infected patients have shown different cytokine responses to the early febrile and defervescence phases. We hypothesized that serial sST2 level changes in DENV patients would correlate with disease severity and coagulopathy in our elderly cohort in the 2015 outbreak.

## Material and methods

### Patients

The records of all DENV-infected patients who, between July 1, 2015, and December 31, 2015, had provided a written informed consent for at least two blood sample analyses were enrolled. The median day with inter-quartile range was 2 [0–4] for the first blood sample and 6 [5–8] for the second blood sample. A DENV diagnosis was confirmed using one or more examinations: positive for serum nonstructural protein 1 (NS1) antigen, dengue IgM antibodies detected using a kit (Bioline Dengue Duo^TM^; Standard Diagnostics, Seoul, Korea), or DENV RNA detected using real-time reverse transcriptase-polymerase chain reaction (RT-PCR) (TIB Molbiol, Lightmix kit; Roche Applied Science, Berlin, Germany). Nineteen volunteers were enrolled as healthy controls.

### Data collection

Demographic data, comorbid diseases, and laboratory data were collected from the patients’ electronic medical records. During hospital admission, DENV severity was evaluated based on the World Health Organization (WHO) 2009 dengue guidelines [1]. Acute Physiology and Chronic Health Evaluation (APACHE) II and Sequential Organ Failure Assessment (SOFA) scores were calculated within the first 24 hour post-admission [21, 22]. Laboratory data were recorded during the admission. The onset day (day 0) of DENV was defined as the day of fever onset. All blood samples were analyzed after the DENV diagnosis had been confirmed. The first sST2 test of residual blood samples was done when a patient presented at an outpatient clinic or an emergency room. The second test was done after a patient was admitted to either a general ward or an intensive care unit (ICU). ΔsST2 was defined as the sST2 level in the second test minus the level in the first test. Heart events included cardiac arrest and elevated cardiac troponin T concentrations. A BSI was defined as any positive blood culture during admission. We excluded patients with only one positive blood culture for skin flora.

### Quantification of soluble ST2 in serum

The serum levels of sST2 were quantified using a kit (Enzyme-Linked Immunosorbent Assay [ELISA] kit: ab100563; Abcam, Cambridge, UK). The lowest concentration in the ELISA standard curves was 1.65 pg/ml for sST2.

### Ethical concerns

This study adhered to the Declaration of Helsinki and was approved by the Human Research and Ethics Committee of National Cheng Kung University (IRB number: B-ER-104-178). All participants were adults. The participants or the next-of-kin of critical ill participants provided a written informed consent and agreed to provide blood samples.

### Statistical analysis

Continuous variables were tested for normal distribution using the Shapiro-Wilk test. Normally distributed variables are expressed as mean ± standard deviation (SD) and compared using Student’s t-test. Non-normally distributed continuous variables are expressed as median plus interquartile range (IQR) and compared using the Mann-Whitney U-test. Categorical data are expressed as proportions and compared using Fisher’s exact test. We computed the Spearman correlation among the first and second sST2 level, the ΔsST2 levels, and the clinical parameters. Receiver operating characteristic (ROC) curve and area under the ROC curve (AUC) analyses were used to evaluate the prognostic ability of ΔsST2 and expressed using 95% confidence intervals (CIs). SPSS 20.0 for Windows (IBM, Armonk, NY) was used to analyze all data. Significance was set at *p* < 0.05 (two-sided).

## Results

### Clinical and laboratory characteristics of patients

**Fig 1:**
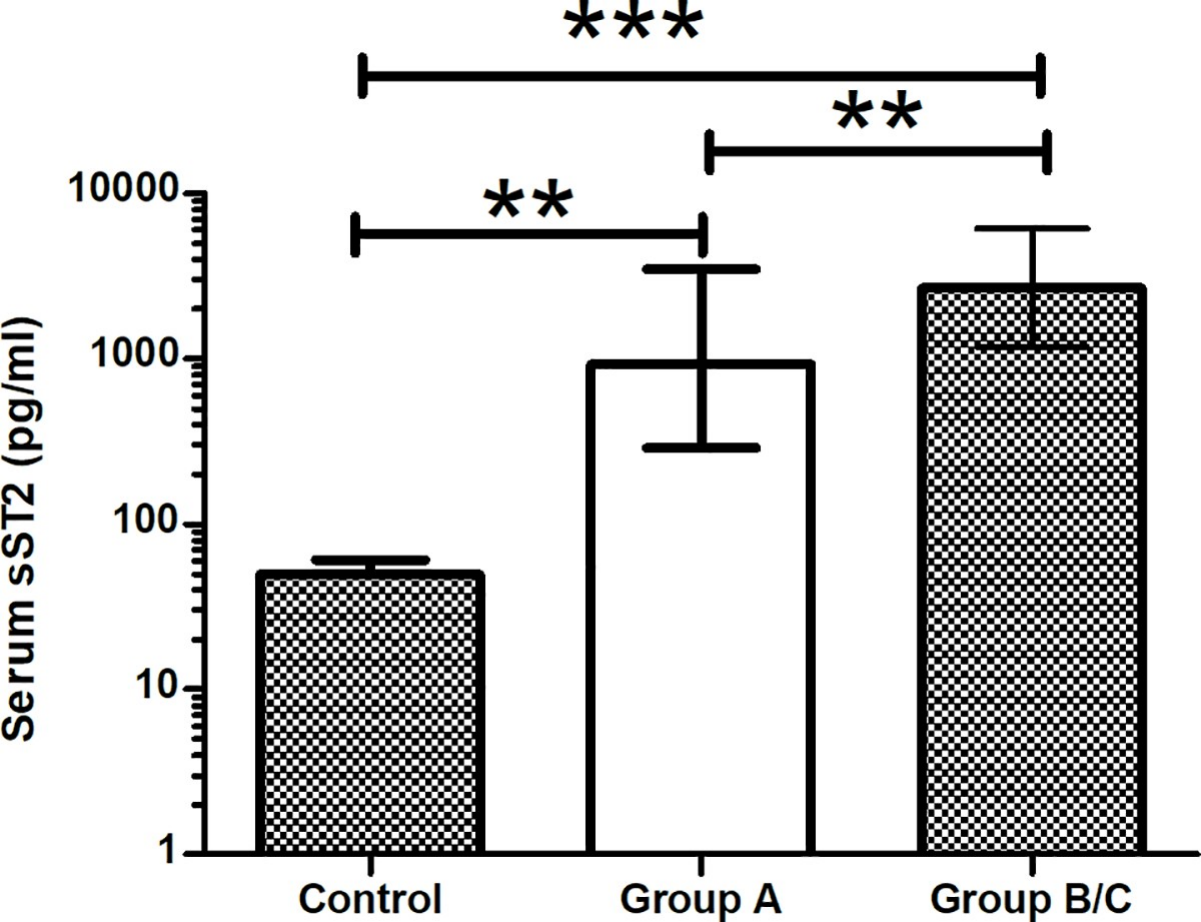
Serum levels of sST2 in different DENV-infected severities. Data are expressed as median (IQR). ***p < 0.001, **p < 0.01.

We initially identified 78 cases of DENV infection: 30 without warning signs, 16 with warning signs, and 32 of severe dengue using WHO 2009 criteria. Serum sST2 levels were significantly positively correlated with the severity of the DENV infection (Fig 1).

Among these 78 cases, 43 cases had paired serum samples and were included in the final analysis. The case fatality rate (CFR) was 44.2% (19/43). The severe dengue cases included 28 cases with warning signs and 15 cases with severe dengue based on the 2009 WHO dengue classification. Median patient age was 75.0 years; IQR was 64.0–78.3 years; and half of them were men.

The SOFA and APACHE II score medians were shown in Table 1. Among our patients, > 60% cases required an ICU admission and most of them needed mechanical ventilation support. Hypertension, diabetes, and chronic kidney disease were the most frequent comorbidities. More than half the patients required a blood transfusion. The non-survivors were older and had significantly higher SOFA and APACHE II scores, ICU admission, usage of mechanical ventilation, and transfusion rather than those of the survivors (Table 1). Significantly more non-survivors than survivors developed heart events and BSIs. Moreover, the non-survivors had longer APTTs and higher aspartate transaminase (AST), and alanine transaminase (ALT) levels than survivors did (Table 2).

**Table 1:** Demographic and clinical characteristics of the survivors and non-survivors with dengue virus infections.

	Overall (N = 43)	Survivors (N = 24)	Non-survivors (N = 19)	*p*
Age, years^a^	75.0 [64.0~78.3]	72.0 [57.5~75.8]	78.0 [71.0~84.0]	0.007
Male	23 (53.5)	14 (58.3)	9 (47.4)	0.547
Disease severity				
SOFA score^a^	11 [5~16]	6 [3~11]	17 [12~20]	<0.001
APACHE II score^a^	19 [15~27]	16 [12~19]	28 [20~39]	<0.001
ICU admission^b^	27 (62.8)	9 (37.5)	18 (94.7)	<0.001
Mechanical ventilation^b^	26 (60.5)	8 (33.3)	18 (94.7)	<0.001
Comorbidity				
Diabetes mellitus	16 (37.2)	8 (33.3)	8 (42.1)	0.752
Hypertension	30 (69.8)	16 (66.7)	14 (73.7)	0.743
Chronic kidney disease	11 (25.6)	5 (20.8)	6 (31.6)	0.495
Coronary artery disease	8 (18.6)	3 (12.5)	5 (26.3)	0.432
Malignancy	8 (18.6)	5 (20.8)	3 (15.8)	1
Dyslipidemia	8 (18.6)	2 (8.3)	6 (31.6)	0.111
Liver disease	5 (11.6)	3 (12.5)	2 (10.5)	1
Transfusion^b^	25 (58.1)	10 (41.7)	15 (78.9)	0.028
Fresh frozen plasma^b^	10 (23.3)	2 (8.3)	8 (42.1)	0.013
Platelets	19 (44.2)	8 (33.3)	11 (57.9)	0.132
Whole blood	14 (32.6)	6 (25)	8 (42.1)	0.329
Packed red cells^b^	19 (44.2)	7 (29.2)	12 (63.2)	0.034
Events during admission				
Heart events^b^	9(20.9)	2(8.3)	7(36.8)	0.022
Bloodstream infections^b^	7(16.3)	1(4.2)	6(31.6)	0.016

Data are presented as median [inter-quartile range] or number of cases (%).

^a^Mann-Whitney U-test.

^b^Fisher’s exact test.

SOFA = sequential organ failure assessment; APACHE II = acute physiology and chronic health evaluation II; ICU = intensive care unit.

**Table 2:** Laboratory parameters of the survivors and non-survivors with dengue virus infections.

	N	Survivors	N	Non-survivors	*p*
PT(seconds)	17	12.2 [11.1–14.4]	16	12.8 [11.8–15.8]	0.087
APTT(seconds)	16	39.3 ± 10.8	16	49.5 ± 12.0	0.017
Hemoglobin (g/dl)	24	12.8 [9.1–13.8]	19	11.7 [10.3–13.1]	0.501
Hematocrit (%)	23	38.1 [27.5–41.7]	18	36.3 [31.6–40.2]	0.655
Platelet (×10^9^/L)	24	99 [28–137]	19	39 [9–125]	0.146
Creatinine (mg/dl)	21	0.99 [0.80–2.19]	19	1.77 [0.97–3.34]	0.250
AST(U/L)	18	113 [36–672]	15	662 [194–1762]	0.020
ALT(U/L)	22	37 [18–181]	19	133 [59–978]	0.030

Data are expressed as mean ± standard deviation or median [interquartile range]

PT = prothrombin time; APTT = activated partial thromboplastin time; AST = aspartate transaminase; ALT = alanine transaminase.

### Changes in sST2 levels in cases of DENV infection

The 1^st^ blood specimens were taken between day -2 and 7. The 1^st^ blood test data were not significantly different between survivors and non-survivors; the 2^nd^ blood test data were, however, significantly higher in non-survivors. Moreover, most non-survivors had an increased sST2 levels, but most survivors had a decreased sST2 level (Table 3). ΔsST2s of the non-survivors were significantly higher than those of the survivors (Table 3).

**Table 3:** Single sST2 concentration and serial changes (ΔsST2) in survivors and non-survivors with dengue virus infections.

	Overall (N = 43)	Survivors (N = 24)	Non-survivors (N = 19)	*p*
The 1^st^ blood specimen taken (Day -2~7)*				
sST2(pg/ml)	2623 [1003~6251]	2562 [1289~5957]	2671 [212~8525]	0.807
The 2^nd^ blood specimen taken (Day 1~14)				
sST2(pg/ml)	1936 [709~8907]	1023 [295~2390]	8907 [4289~36000]	<0.001
Serial changes				
ΔsST2(pg/ml)	-275 [-2413~3702]	-1354 [-5210~-432]	3406 [0~8693]	<0.001

Data are expressed as median [interquartile range]. Day 0 is defined as the time of fever onset

*day -2 is two days prior to fever onset; sST2 = soluble ST2; ΔsST2 = the difference between (change in: Δ) two measurements of the sST2.

### Correlations between ΔsST2 levels and clinical parameters

**Fig 2:**
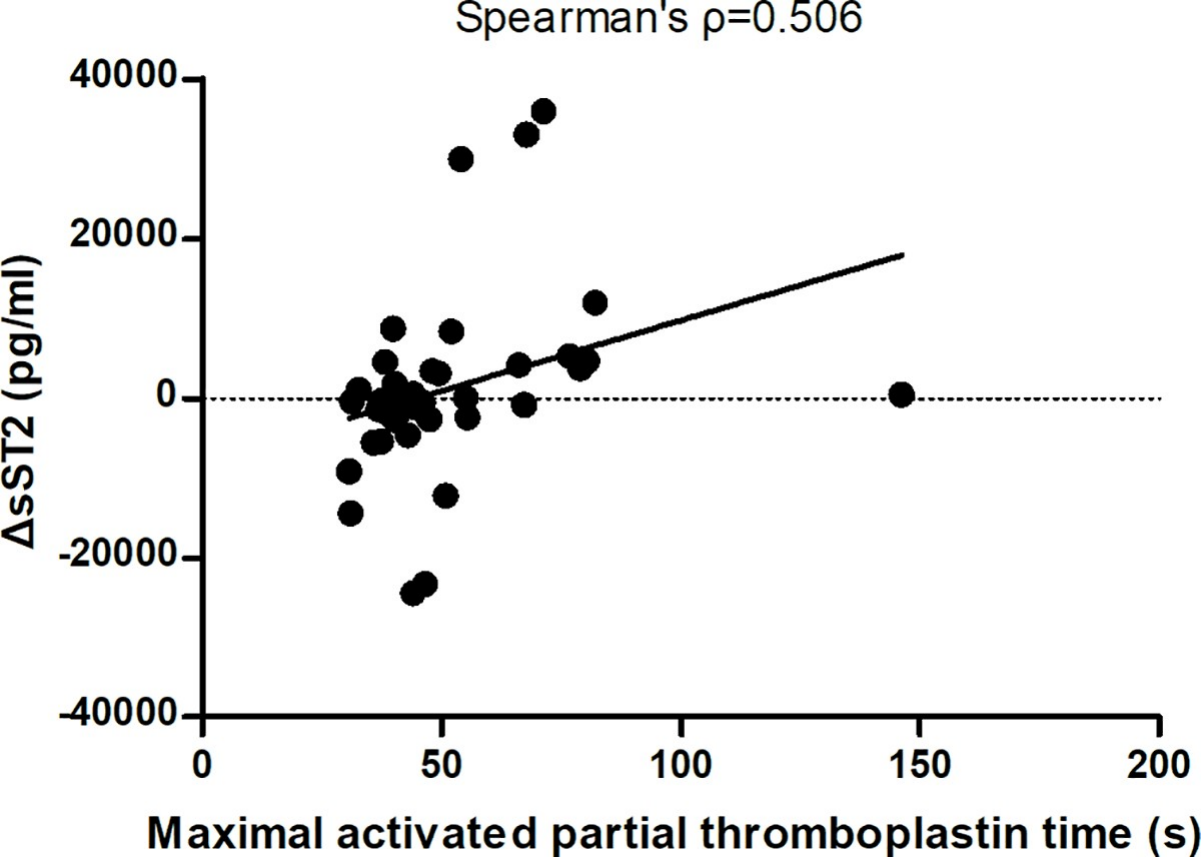
ΔsST2 was positively correlated with maximal activated partial thromboplastin time (APTT).

**Fig 3:**
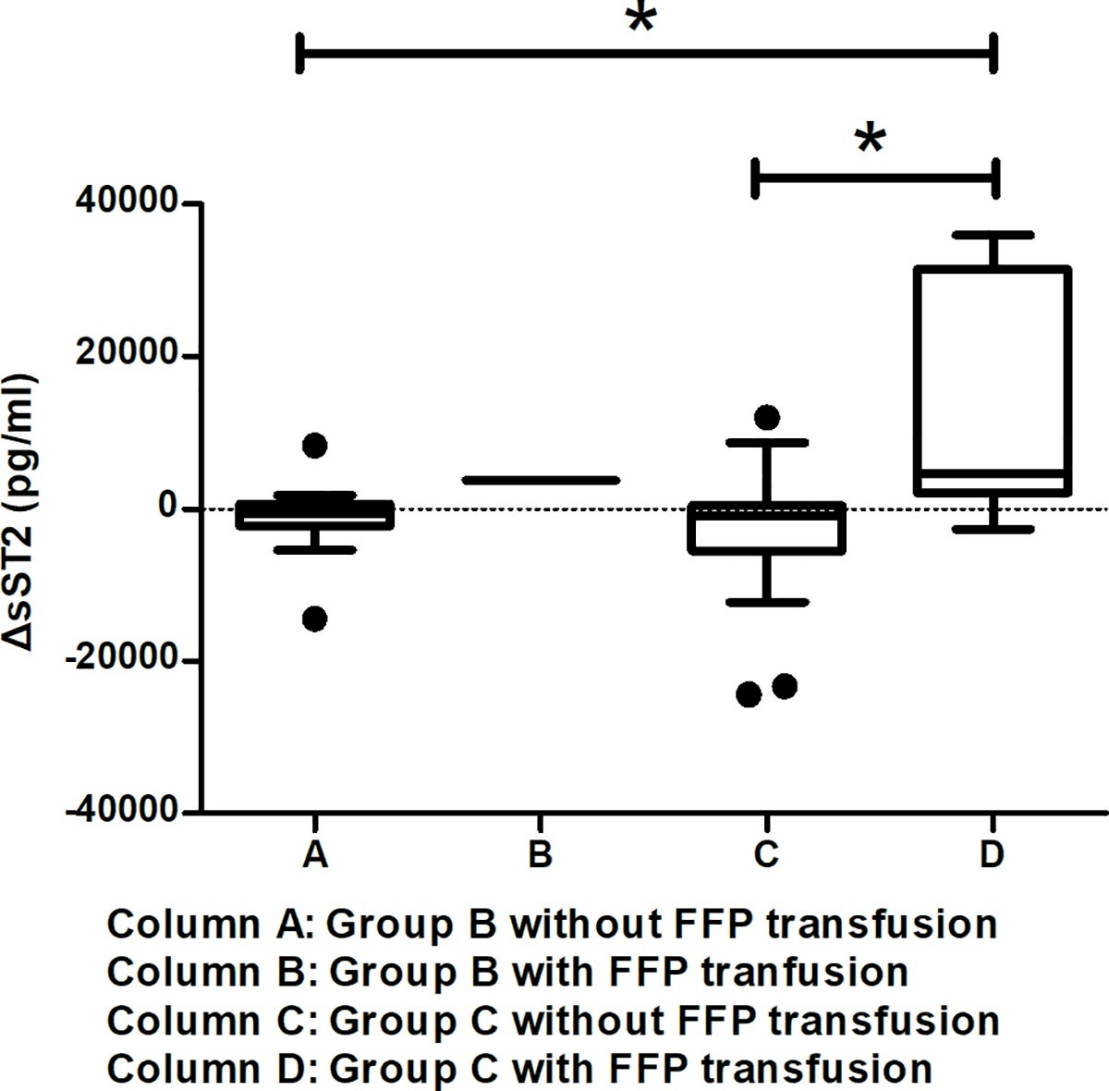
The box plot of ΔsST2 based on DENV-infection severity with and without a fresh frozen plasma (FFP) transfusion. *p < 0.05.

The correlations of the available clinical parameters were calculated with the first sST2, and second sST2 test data, and with ΔsST2 levels. Positive BSIs were significantly associated with the second sST2 levels (Table 4). Except for the minimal platelet count, most clinical variables were not correlated with the first sST2 levels. ΔsST2 levels were positively correlated with disease severity (e.g., SOFA and APACHE II scores), maximal APTT, maximal prothrombin time (PT), maximal AST, maximal ALT, and FFP transfusions (Table 4 & Fig 2). Most patients who required an FFP transfusion had increased sST2 levels: ΔsST2 levels in patients with an FFP transfusion vs. without an FFP transfusion: 4604 [2776~30712] vs. -605 [-3645~463], *p* = 0.011 (Fig 3). Seven patients tested positive for BSIs during hospital admission (Table 5). Four of them developed significant bacteremia within the first week of ICU admission, and all four died. Five of 14 (35%) elderly patients with increased serum sST2 did have BSIs. Five of seven (71.4%) elderly patients with BSIs had increased serum sST2. The detail laboratory data of these patients are listed in S2 Dataset.

**Table 4:** Correlation matrix between sST2, its changes and clinical parameters.

	N	First sST2	Second sST2	ΔsST2
**Maximal Body temperature**	43	-0.244	-0.130	0.030
**Minimal WBC**	43	-0.045	0.090	-0.011
**Maximal WBC**	43	-0.082	0.270	0.267
**Minimal Hematocrit**	43	-0.118	-0.349*	-0.182
**Minimal platelet count**	43	-0.318*	-0.509*	-0.266
**Maximal PT**	40	0.193	0.669*	0.498*
**Maximal APTT**	39	-0.071	0.425*	0.506*
**Maximal Creatinine**	42	0.268	0.533*	0.247
**Maximal AST**	43	0.193	0.622*	0.450*
**Maximal ALT**	43	0.223	0.560*	0.328*
**Liver disease**	43	0.044	0.000	-0.026
**Bloodstream infections**	43	0.272	0.417*	0.251
**Heart event**	43	-0.113	0.115	0.138
**APACHE II**	43	-0.090	0.558*	0.564*
**SOFA**	43	0.128	0.676*	0.527*
**Transfusion**	43	0.173	0.521*	0.342*
** FFP transfusion**	43	0.007	0.533*	0.517*
** Packed red cell transfusion**	43	0.176	0.544*	0.400*
** Platelet transfusion**	43	0.045	0.507*	0.387*
**Outcome**	43	-0.038	0.586*	0.615*

* *p* < 0.05

N = case number; WBC = white blood cell; PT = prothrombin time; APTT = activated partial thromboplastin time; AST = Aspartate aminotransferase; ALT = alanine aminotransferase; APACHE II = acute physiology and chronic health evaluation II; SOFA = sequential organ failure assessment; FFP = fresh frozen plasma; sST2 = soluble ST2; ΔsST2 = the difference between (change in: Δ) two measurements of the sST2.

**Table 5:** Characteristics and timing of blood samples in the cases with bloodstream infections.

**Case no.**	**Age(year), sex**	**Comorbidity**	Day of ER visit*	**Antibiotics in ER**	Day of blood culture*	**Isolated pathogen**	**Day of 1**^**st**^ sST-2*	**1**^**st**^ **sST-2 level**	**Day of 2**^**nd**^ sST-2*	**2**^**nd**^ **sST-2 level**	**Outcome**
**#1**	71, F	HTN,CKD	4	cefotaxime	9	*Candida tropicalis*	4	8252	8	12914	Died
**#2**	73, F	None	0	No	3	*Klebsiella pneumonia*, *Proteus mirabilis*	1	10048	5	10570	Died
**#3**	82, M	DM, HTN, CKD, CAD	2	No	7	*Klebsiella pneumonia*	4	7678	6	10771	Died
**#4**	85, F	DM, HTN	2	No	2	*Escherichia coli*, *Streptococcus gallolyticus* Subspecies *pasteurianus*	2	2671	5	14667	Died
**#5**	86, F	HTN,CKD,DM	3	cefuroxime	3&5	*Streptococcus salivarius*, *Escherichia coli*	3	587	6	4289	Died
**#6**	76, M	HTN, liver disease	5	cefotaxime	8	*Candida tropicalis*	5	65358	11	47297	Died
**#7**	75, F	HTN, CAD, DM	3	cefotaxime	27	*Elizabethkingia meningoseptica*	3	5188	8	2541	Survived

* Day 0 is defined as the time of fever onset; HTN = hypertension; CKD = chronic kidney disease; CAD = coronary artery disease; DM = diabetes mellitus.

### ΔsST2 levels predicted deadly DENV-infections

**Fig 4:**
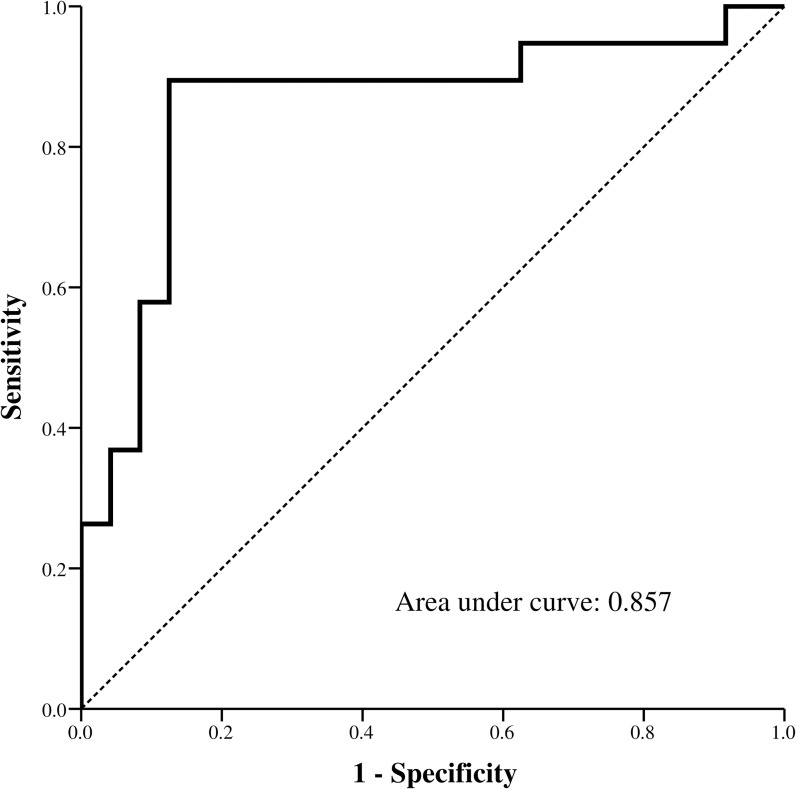
Receiver operating characteristic (ROC) curve analysis presentation of ΔsST2 to predict fatality in DENV infection cases.

The ROC curve for ΔsST2 levels predicted death, and the AUC value for ΔsST2 levels was 0.857 (CI: 0.731–0.983) (Fig 4).

## Discussion

Our most important findings were that ΔsST2 levels were positively correlated with DENV-infection severity scores, maximal APTT, maximal PT, maximal AST, maximal ALT, and FFP transfusions, and that increased sST2 positively predicted death. ΔsST2 levels were not correlated with heart events through the admission course. Serum sST2 levels are elevated in patients with autoimmune disease, burn injuries, and sepsis [23–25] and especially in the cardiovascular system they are correlated with disease severity and patient death [26, 27]. This discrepancy might be related to the low incidence of heart events in our cohort. The other cytokine such as IL-6, TNFα, response to early febrile and defervescence phases of DENV-infection varied significantly and even led to contradictory conclusions [16]. Because there was a significant difference in the second sST2 test, the trend of sST2 levels is more important than a single observation for predicting death in elderly patients with DENV-infection. Thus, serial sST2 levels are more important for showing correlations between sST2 than single levels are [23].

Monitoring the trend of sST2 levels to predict fatality of elderly dengue could be supported by previous researches reported in other age group [12, 18], for instance, Becerra and Guerrero et al. reported that sST2 levels were elevated in children and young adults with dengue fever. Serum sST2 levels sampled on days 3–6 were significantly different between healthy controls and patients with severe dengue, which suggested that sST2 might be a useful marker of the severity of DENV infection [18]. We confirmed these are also valid predictors in our elderly cohort and verified an association between patient outcome and changes in sST2 levels. Elderly DENV infection survivors had lower ΔsST2 levels than did the non-survivors. The AUC for ΔsST2 levels that predicted dengue fatality was 0.857. Thus, ΔsST2 might be a viable predictor comparable to APTT, as previously reported [4]

Our findings indicate that ΔsST2 levels correlate with the severity of coagulopathy; however, the mechanism is unclear. This is the first study that analyzes the association between ΔsST2 levels and coagulopathy in DENV infection. The severity of DENV infection has been associated with the degree of coagulation and fibrinolysis activation induced by DENV [28]. ΔsST2 level correlates with DENV infection fatality and is proportional to the coagulopathy. It might play a pivotal role in the coagulopathy mechanism of DENV infections. One study [29] claimed that IL-33 expression decreased in patients with immune thrombocytopenia. IL-33 increases cell surface tissue factor, a major trigger of coagulation, and it significantly reduces the coagulation time of human whole blood and plasma samples [30]. Elevated ST2 levels plus normal or low IL-33 expression might interfere with coagulation in cases of DENV infection, as they do in cases of immune thrombocytopenia. This cytokine pattern (high ST2 level plus normal IL-33) inversely correlated with thrombocytopenia was confirmed in a young DENV-infected population [18], and it might be the cellular mechanism between sST2 and coagulopathy in DENV infection. Our medical college colleagues also have hypothesized that pathogen-triggered autoimmunity (molecular mimicry) involves cross-reactivity of DENV with human endothelial cells, platelets, and coagulatory molecules [31]. Whether serum ST2 and IL-33 are associated with pathogen-induced autoantibodies cross-reacting with coagulopathy molecules remains uncertain.

Few studies of DENV-infection and concurrent bacteremia have been reported; however, DENV-infections with concurrent BSIs are significantly more deadly than those without them are [32]. We previously reported [4] that 9.3% (7/75) of ICU patients had bacteremia within 48 hours after they had been admitted to the ICU. Although the predictors of concurrent bacteremia are unknown, clinical suspicion in critical cases with elderly patients is warranted.

IL-1R and IL-1Ra (IL-1R antagonist) are important modulators of sepsis and sepsis-related immune dysfunction. IL-1Ra was also a good predictor of clinical outcomes in cases of febrile neutropenia because, like C-reactive protein and procalcitonin, it predicts severe sepsis in the early stages [33]. sST2, a derivative of IL-1R, might also be able to predict BSIs in elderly patients with DENV-infection. Six of our seven initially critically ill patients with a BSI died. Four of them had early-onset BSIs and had not been treated with antibiotics during their first medical visit. These patients with a BSI did not have leukopenia in the critical phase, and ΔST2 level increased in these patients. DENV-infected patients without leukopenia but with increased ST2 levels in the critical phase might have concurrent sepsis.

### Limitations

This study has limitations. First, this is a retrospective study and the serum samples came from the residual blood of routine laboratory tests. Thus, we cannot perform the sST2 measurement in a pre-specified timing. The optimal timing of sST2 testing needs further study. Second, we had an inclusion selection bias. Patients with a mild DENV-infection had only one chance for a blood test during the first visit then they were discharged. Patients with severe diseases had paired serum samples. Third, we did not complete testing for IL-33 in all patients because the levels of IL-33 were not correlated with the clinical outcomes in our preliminary survey (S3 Dataset and S1 Fig). This finding is compatible with a pediatric study [18].

### Conclusions

Serum sST2 levels were higher in the elderly patients with DENV-infection than the normal controls. Serial serum sST2 changes reflected infection severity in the elderly (≥ 65 years), which has not been previously reported. Serum sST2 is an important coagulopathy modulator in severe DENV infections. Five of 14 elderly patients with elevated serum sST2 did have BSIs.

## Supporting information

S1 Checklist STROBE checklist. (DOCX)

S1 Dataset The Dataset of this study. (XLS)

S2 Dataset The Dataset of the cases with BSIs. (XLSX)

S3 Dataset The IL-33 levels in health controls and DENV-infected patients. (XLSX)

S1 Fig The IL-33 levels are not different in health controls and different groups of DENV-infected patients. Data are expressed as median (IQR). (Kruskal-Wallis test, *p* = 0.129)
(TIF)
