# Supplementary figures and images for: Dynamic changes of soluble ST2 levels predicted fatality and were involved in coagulopathy in dengue fever in the elderly
Source: PLoS Negl Trop Dis. 2019 Dec 26;13(12):e0007974. doi: 10.1371/journal.pntd.0007974 (PMC6948823; doi:10.1371/journal.pntd.0007974)

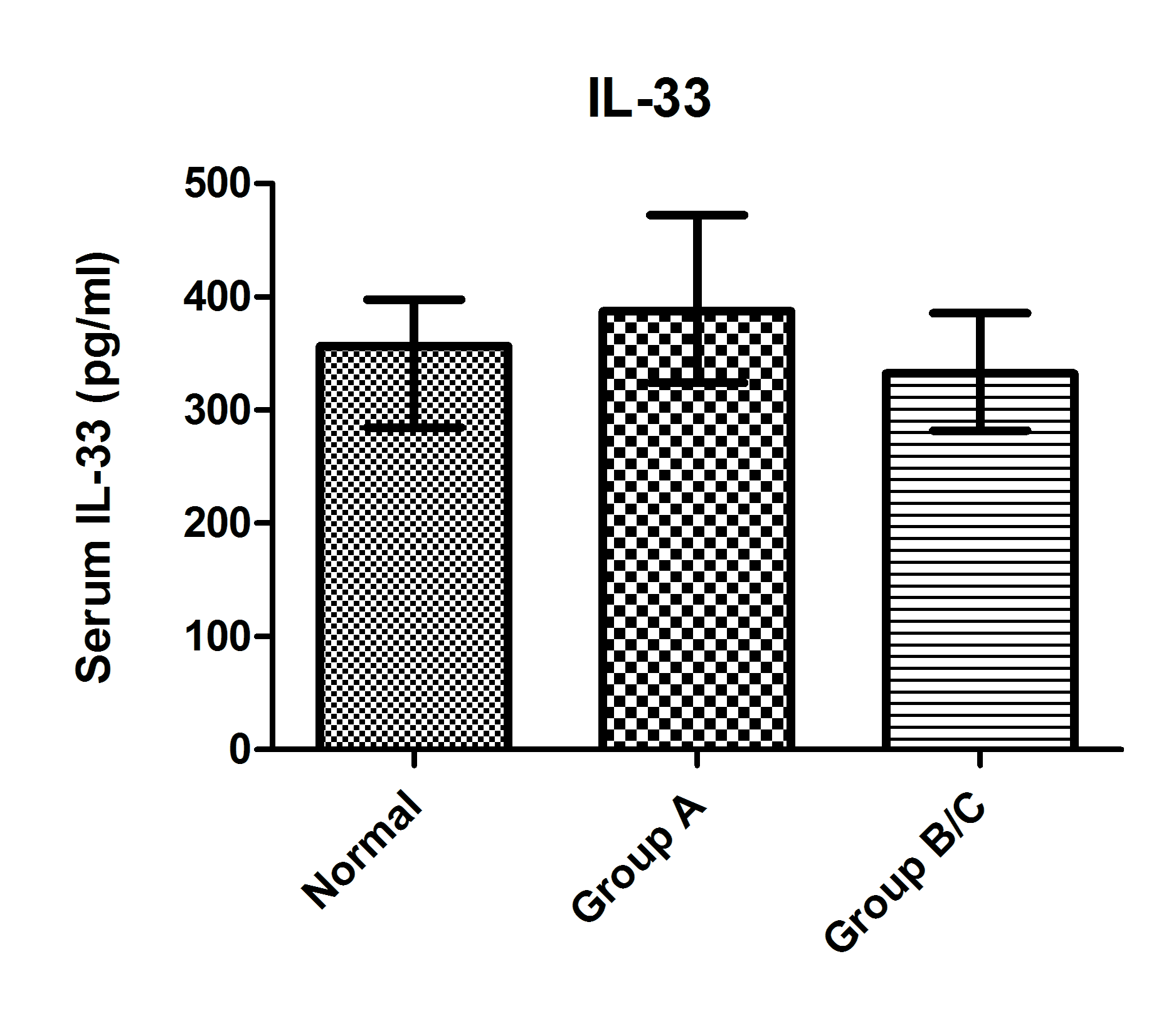

Supplement: S1 Fig — Data are expressed as median (IQR). (Kruskal-Wallis test, p = 0.129) (TIF) [file pntd.0007974.s005.tif]
